# Assessment of Eurasian lynx reintroduction success and mortality risk in north-west Poland

**DOI:** 10.1038/s41598-022-16589-2

**Published:** 2022-07-20

**Authors:** Jakub Skorupski, Magdalena Tracz, Maciej Tracz, Przemysław Śmietana

**Affiliations:** 1 Institute of Marine and Environmental Sciences, University of Szczecin, 71-415 Szczecin, Poland; 2 West Pomeranian Nature Society, 71-415 Szczecin, Poland; 3 Polish Society for Conservation Genetics LUTREOLA, 71-784 Szczecin, Poland

**Keywords:** Conservation biology, Restoration ecology

## Abstract

Despite recent local reintroductions of the Eurasian lynx *Lynx lynx* in central and north-eastern Poland, the increase in its population was not followed by its westward expansion. To address this problem and restore the lynx population in north-western Poland, 61 captive-born individuals of Baltic population origin were released in the province of Western Pomerania in 2019–2021. Prior to their release, all the animals underwent an adaptation training phase. They were then set free according to a hard-release protocol and subsequently monitored by means of GPS telemetry. In order to assess the short-term reintroduction success, the survival and causes of death of the released individuals were studied as a function of sex, age, training time, and release time and place. The overall survival rate was 71.15%, the median survival time 202 days. Most mortality, due to environmental factors, i.e. scabies (> 200 days) or traffic collisions (< 200 days), was recorded during the first 300 days following release. Age, year of release and training time were significantly related to survival, indicating that the older the lynx was when released, the better its survival changes. In contrast, the longer the training time, the poorer were the chances of survival. There was no evidence of any effect of sex, month of release or place of release. Based on these results, recommendations were made for the planning of further releases and measures to manage the restored population.

## Introduction

The Eurasian lynx *Lynx lynx*, L. 1758 (henceforth—lynx) is the largest felid in Europe and the third largest representative of the order Carnivora on the continent, after the brown bear and the grey wolf^1,2^. The species used to be widely distributed throughout Europe, but by the early twentieth century it had become extirpated from most of the areas it had occupied in western and central Europe. By that time, the lynx’s range had contracted to a historical minimum^4,5^. The rapid decline in its populations was caused by deforestation, loss of prey, habitat fragmentation and overhunting^3–6^. At present, the European range of the lynx covers approximately 813,400 km^2^, i.e. the area occupied by five autochthonous populations in Fennoscandia (Scandinavian and Karelian), the south-eastern Baltic catchment area, the Carpathians and Balkans, as well as six restored populations in central and western Europe (Dinaric, Bohemian-Bavarian, Alpine, Jura, Vosges-Palatinian and Harz Mountain)^6,7^. At present, lynx are found in 23 European countries^6^. Some 9000–10,000 individuals are estimated to be persisting in the wild in continental Europe, excluding Russia and Belarus^7^.

The situation of the lynx over the centuries in Poland is a good illustration of its predicament in central Europe: deteriorating ecological conditions for its existence coupled with negative human attitudes led to its disappearance, first from the lowlands and later from mountain areas, by the end of the nineteenth century^2,8,9^. Already by the late eighteenth century the lynx had become extinct west of the River Vistula, and further disappearances took place farther east in subsequent decades^9^. The lynx was eradicated from north-western Poland between the fifteenth and eighteenth centuries^9^. By the mid-nineteenth century, therefore, the Baltic and Carpathian populations had become isolated from each other^9^. The situation worsened over the next hundred years, so that by the start of the Second World War only two populations remained—one in the Białowieża Primeval Forest and the other in the Carpathian Forest^9^. In the post-war years, however, the situation started to improve, and the lynx’s population in Poland slowly rose^9–11^, culminating in some 600 animals in the 1980s^8^. Thereafter, it started to decline, and by the beginning of the twenty-first century the population had fallen to an estimated 200 individuals, scattered in local natural populations in north-eastern and south-eastern Poland, with just a few dispersing animals occasionally recorded in north-western Poland and the Sudety Mountains^5,10–12^. At the end of the 2000s, there were an estimated 285 lynx in the country^13^. The approximate extent of the lynx’s current range in Poland is 10,800 km^2^ ^12^, but this population increase has not been matched by a corresponding range expansion in the country, even after it had been granted strict legal protection in 1995^11^.

At the European scale, the decline of the lynx population and the shrinkage of its distribution were both arrested in the mid-twentieth century^5^. A combination of several species-enhancing factors, including containment of large-scale deforestation, improvement of the situation of the roe deer population—the most important prey species, a significant reduction in the scale of lynx hunting, as well as granting the species legal protection, has enabled the lynx to recolonize parts of its former range^3,5,6^. Consequently, the overall situation of the species has much improved recently, which is reflected in its categorization as “least concern” (worldwide) by the International Union for Conservation of Nature (IUCN) Red List of Threatened Species^14^. Nevertheless, some local populations in Europe are endangered because of demographic instability, limited ecological connectivity and the consequent restricted gene flow, as well as anthropogenic mortality (i.e. illegal kills)^6,15–20^.

In order to further improve the lynx’s situation in Europe and restore its local populations, 15 reintroduction projects, involving over 170 individual animals, were carried out across eight countries from the 1970s to the 2000s^3^. Five of these attempts have been successful^3^. Three reintroduction programmes have so far been carried out in Poland. In 1993–2000 a small, viable population was formed from captive-born individuals in central Poland in the Kampinos National Park^8,21^. Between 2003 and 2006 another reintroduction of captive-born lynx took place in the Pisz Forest in north-eastern Poland^3^. In 2012–2015, a combination of zoo-originated and translocated wild animals were released in the Pisz Forest and in the neighbouring Napiwoda-Ramuki Forest^22^. The greatest potential barriers to the lynx’s range expansion in Poland, mainly towards the west, are habitat fragmentation by non-forested and urban areas, and transportation infrastructure^23,24^. In addition, the genetic variability of the autochthonous population in north-eastern Poland is lower than that of the populations in Estonia and Latvia; the former being isolated, it suffers from limited gene flow. This implies the need for further active conservation measures to restore ecological connectivity, support natural expansion and increase the viability of native and reintroduced populations^18,25,26^.

The current reintroduction project has been conducted since 2017 by the West Pomeranian Nature Society, in cooperation with the Mammal Research Institute of the Polish Academy of Sciences in Białowieża, the Cultural Center in Mirosławiec and the World Wide Fund for Nature (WWF) Poland. The project aims to restore the population of the lowland lynx in north-western Poland and to ensure appropriate conditions for its development^27^.

The very limited westward expansion, if any, of the lynx into the western lowlands of Poland, and often the lack of appropriate monitoring and evaluation of reintroduction programmes justify the properly planned, continuous tracking of the fate of the reintroduced animals. This will enable the short- and long-term evaluation of current reintroductions and inform future projects of this kind^3,8,11^. The IUCN defines post-release monitoring as “the means to measure the performance of released organisms against objectives, to assess impacts, and provide the basis for adjusting objectives or adapting management regimes or activating an exit strategy”^28^. Of key importance is the assessment of demographic performance, including the monitoring of population growth and spread in order to estimate individual survival, reproduction and dispersal^28^. In addition, such monitoring serves to coordinate conservation measures for the species, including the ongoing management of its populations, in the whole region.

To address these issues, we assess here the short-term reintroduction success of the “Return of the Lynx to north-western Poland” project, based on post-release survival indicators. The aim of this study was to evaluate the longevity and adaptation to life in the wild of the released animals, assuming that greater survival (and independence of humans) is expressed by the effectiveness of hunting, and subsequent mating that ends in parturition and the rearing of kittens. The effects of sex, age, time spent in captivity, release date and location were determined. The causes of lynx deaths were also reported and analysed. Based on the conclusions drawn, recommendations were made for the pre-release, as well as for further stages of reintroduction and management measures of the restored lynx population in north-western Poland. All the analyses were performed for the data as of 30 September 2021, as the reintroduction project is being continued. We hypothesized that traffic collisions were the main mortality factor^29^. Data on post-release migration and spatial distribution, as well as reproductive success will be published in a separate paper.

## Results

### Fate of the released animals

Sixty-one Eurasian lynx—35 males and 26 females—were released between January 2019 and July 2021. Thirty-seven of these 61 animals, including one female caught after release and re-released in another location, were alive in the wild as of 30 September 2021. Field observations, conducted as part of the post-release monitoring, indicate that these animals have settled well in the wild, are efficient hunters and are only very occasionally sighted by people.

**Figure 1:**
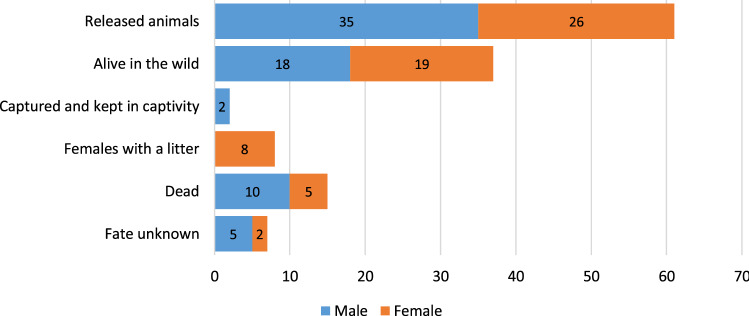
Summary of the fate of Eurasian lynx (N = 61) released during a reintroduction project in north-western Poland, as of 30 September 2021.

Two males were caught soon after being released and were placed permanently in the breeding centre. In one case this was due to the need for treatment, and in the other because the animal would persistently remain near human settlements, sometimes even entering farmhouses. In the period in question, ten certain cases of reproduction of released individuals were recorded: six females had one litter and two others gave birth twice in two consecutive years. Fifteen of the released animals died in the wild. The fate of seven others is not known because there was no GPS contact (telemetry collar malfunction or, in one case, its loss). Figure 1 summarizes the fate of all the individuals released, separately for males and females, while Table 1 sets out details of the individuals, i.e. sex, date of birth, age, time spent in the adaptation enclosure and release centre, date and place of release, date of death, survival time and fate. The mean age of the released males is 36.6 months, and females 35.1 months, while the mean training time of males and females is 566.1 and 532.5 days, respectively.

**Table 1:** Characteristics of Eurasian lynx reintroduced in north-western Poland in January 2019–July 2021 (age—as of 30 September 2021 for live animals; survival time—number of days from release until death was confirmed or until 30 September 2021 for live animals; fate (status as of 30 September 2021): C—caught and placed in an enclosure, A—alive, L—a female that had a litter, D—dead, with cause of death marked in parentheses as unknown (U), environmental factors/scabies (S) or road collision (C), U—status unknown; n/a—not applicable; the animals caught soon after release and placed in the breeding centre are marked in bold; the animals caught soon after release and re-released in another location are marked in italics).

No.	Sex	Date of birth	Age (months)	Time spent in enclo-sure/training time (days)	Release date	Place of release	Date of death (dead animal found)	Survival time (days)	Fate
**1**	**Male**	**05.2019**	**28**	**498**	**26.03.2020**	**Mirosławiec**	**n/a**	**553**	**C**
**2**	**Male**	**04.2019**	**29**	**416**	**25.06.2020**	**Drawsko**	**n/a**	**462**	**C**
3	Female	05.2016	65	530	04.09.2019	Dłusko	n/a	757	A
4	Female	06.2013	n/a	1595	19.05.2019	Dłusko	n/a	n/a	U
5	Male	05.2012	114	2383	29.03.2019	Dłusko	n/a	916	A
6	Male	05.2016	65	947	23.01.2019	Dłusko	n/a	981	A
7	Male	05.2017	31	642	09.02.2019	Dłusko	31.12.2019	325	D_(S)_
8	Male	05.2018	41	280	16.04.2019	Dłusko	n/a	898	A
9	Male	05.2018	34	280	16.04.2019	Dłusko	13.02.2021	669	D_(S)_
10	Female	05.2010	132	3216	28.02.2019	Dłusko	11.03.2021	742	L, D_(S)_
*11*	*Female*	*05.2017*	*54*	*764*	*04.06.2019*	*Dłusko, Mirosławiec*	*21.10.2020*	*505*	*L, D*_*(U)*_
12	Male	05.2018	15	304	28.04.2019	Dłusko	03.08.2019	97	D_(C)_
13	Male	05.2018	41	323	02.04.2019	Dłusko	n/a	912	A
14	Male	06.2017	52	708	21.05.2019	Mirosławiec	n/a	863	A
15	Male	05.2018	23	386	04.06.2019	Mirosławiec	12.03.2020	282	D_(U)_
16	Female	05.2017	53	785	29.12.2020	Mirosławiec	n/a	275	A, L
17	Male	05.2018	23	392	18.07.2019	Mirosławiec	23.03.2020	249	D_(S)_
18	Male	06.2017	28	708	21.05.2019	Mirosławiec	29.09.2019	131	D_(U)_
19	Male	06.2018	40	396	30.07.2019	Mirosławiec	n/a	793	A
20	Male	06.2018	40	396	07.08.2019	Mirosławiec	n/a	785	A
21	Male	06.2015	76	598	07.08.2019	Dłusko	n/a	785	A
22	Female	05.2014	89	593	20.10.2019	Dłusko	n/a	711	A, L
23	Female	05.2018	41	469	21.08.2019	Drawsko	n/a	771	A
24	Female	05.2019	28	22	04.09.2019	Dłusko	n/a	757	A
25	Male	05.2019	n/a	132	26.03.2020	Mirosławiec	n/a	n/a	U
26	Male	05.2019	13	132	03.04.2020	Dłusko	06.06.2020	64	D_(U)_
27	Female	05.2019	12	132	03.04.2020	Dłusko	09.05.2020	36	D_(U)_
*28*	*Female*	*03.2018*	*43*	*581*	*29.03.2020*	*Mirosławiec, Dłusko*	*n/a*	*550*	*A*
29	Male	03.2018	24	581	17.10.2019	Drawsko	03.03.2020	138	D_(C)_
30	Male	05.2017	n/a	892	25.10.2019	Mirosławiec	n/a	n/a	U
31	Female	05.2018	19	563	29.11.2019	Dłusko	04.12.2019	5	D_(C)_
32	Male	05.2018	41	563	29.11.2019	Mirosławiec	n/a	671	A
33	Male	05.2014	89	2108	09.03.2020	Mirosławiec	n/a	570	A
34	Female	04.2019	29	331	25.03.2020	Mirosławiec	13.10.2020	202	D_(C)_
35	Male	04.2019	29	331	21.03.2020	Mirosławiec	n/a	558	A
36	Male	04.2019	29	331	31.03.2020	Drawsko	n/a	548	A
37	Male	05.2019	n/a	307	10.04.2020	Mirosławiec	n/a	n/a	U
38	Male	05.2019	20	316	05.05.2020	Dłusko	26.12.2020	235	D_(S)_
39	Male	04.2019	n/a	416	24.06.2020	Dłusko	n/a	n/a	U
40	Male	04.2019	29	416	04.07.2020	Mirosławiec	n/a	453	A
41	Male	05.2019	n/a	399	26.06.2020	Mirosławiec	n/a	n/a	U
42	Male	05.2019	28	399	07.07.2020	Dłusko	n/a	450	A
43	Female	05.2019	28	412	24.08.2020	Dłusko	n/a	402	A, L
44	Female	05.2019	28	412	02.10.2020	Drawsko	n/a	363	A, L
45	Male	05.2019	28	426	30.07.2020	Mirosławiec	n/a	427	A
46	Male	05.2018	30	791	22.07.2020	Drawsko	05.10.2020	75	D_(U)_
47	Male	05.2019	28	434	29.08.2020	Drawsko	n/a	397	A
48	Female	05.2019	28	434	14.11.2020	Dłusko	n/a	320	A, L
49	Female	05.2019	28	456	10.09.2020	Mirosławiec	n/a	385	A
50	Female	05.2019	28	456	20.10.2020	Mirosławiec	n/a	345	A, L
51	Male	06.2020	15	204	25.01.2021	Mirosławiec	n/a	248	A
52	Female	06.2020	15	204	14.01.2021	Mirosławiec	n/a	259	A
53	Female	05.2020	n/a	307	04.03.2021	Dłusko	n/a	n/a	U
54	Female	05.2020	16	294	21.06.2021	Drawsko	n/a	101	A
55	Male	05.2020	16	294	07.03.2021	Mirosławiec	n/a	207	A
56	Female	05.2020	16	294	22.05.2021	Mirosławiec	n/a	131	A
57	Female	05.2019	27	399	30.06.2021	Mirosławiec	n/a	26	A
58	Female	05.2020	16	305	05.05.2021	Mirosławiec	n/a	148	A
59	Female	05.2020	16	305	10.05.2021	Drawsko	n/a	143	A
60	Female	05.2020	16	411	27.06.2021	Drawsko	n/a	95	A
61	Female	05.2020	16	411	23.06.2021	Mirosławiec	n/a	99	A

### Survival rate

The overall survival rate (covering the period from the first release, i.e. 23.01.2019, to 30.09.2021) of the released animals (N = 52) was 71.15%; the figure for males (N = 28) was 64.29% and for females (N = 24) 79.17%. There was no significant difference between the sexes in this respect (chi-squared test, *P* = 0.24). Assuming that the fate of lynx from which telemetry data are no longer received is known, this parameter would be 74.58% for all released individuals (N = 59), 69.7% for males (N = 33) and 80.77% for females (N = 26) if these animals were still alive, or 62.71% for all animals (N = 59), 54.55% for males (N = 33) and 73.08% for females (N = 26) if these animals were dead.

**Figure 2:**
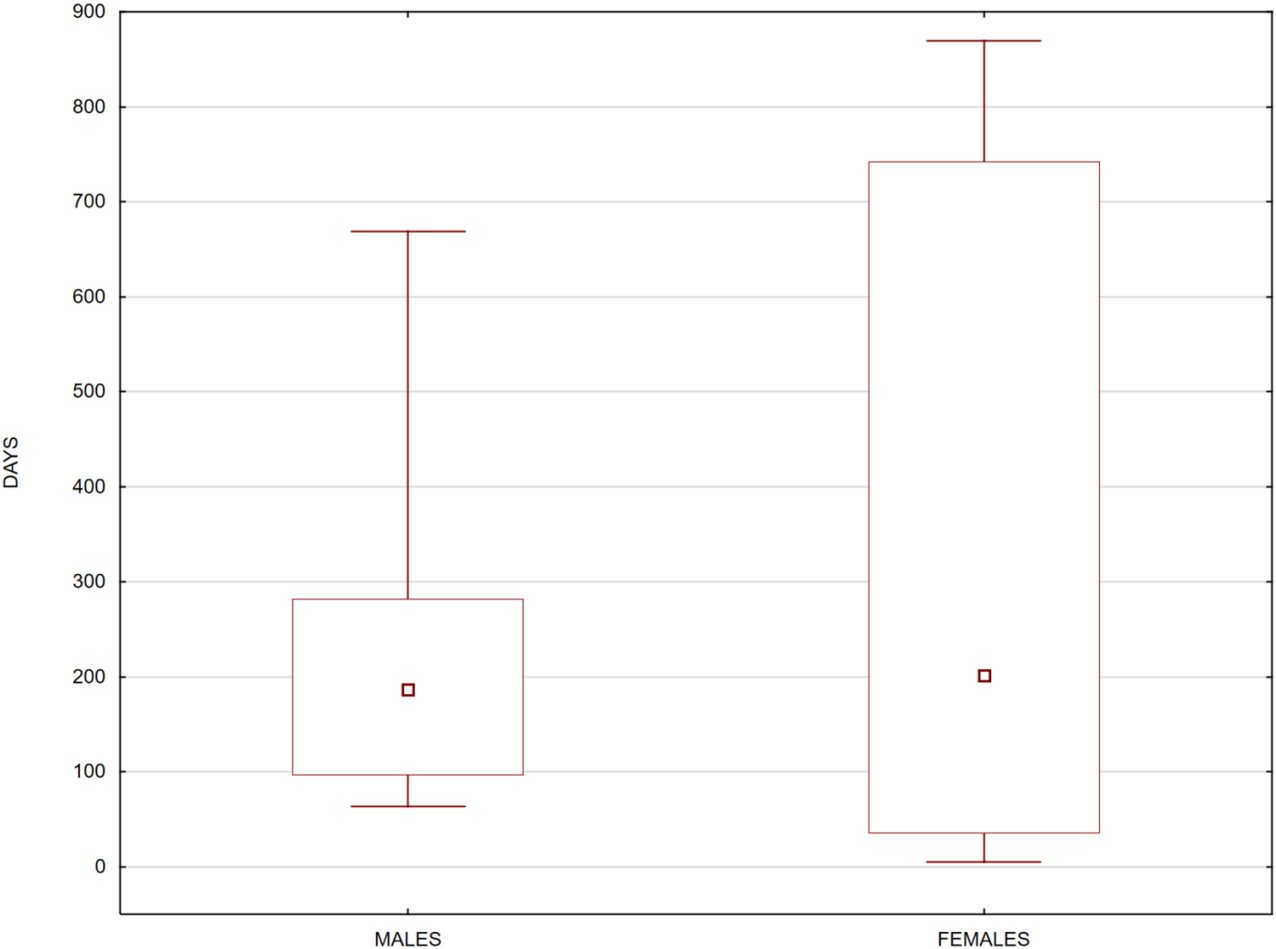
Box-and-whisker plots of male and female (N = 15) life expectancy, expressed as the number of days from the date of release to the date of confirmed death in the wild (the square inside each box indicates the median value, the edges of the box are the 25th and 75th percentiles, and the whiskers extend to the most extreme points).

The median survival time (N = 15) was 202 days (males—186.5 days, females—202 days). No significant differences were found between the sexes (Mann–Whitney *U*-test for comparisons of median values, *P* = 0.95), but there was much greater variability in life expectancy in the wild for females (Fig. 2).

**Figure 3:**
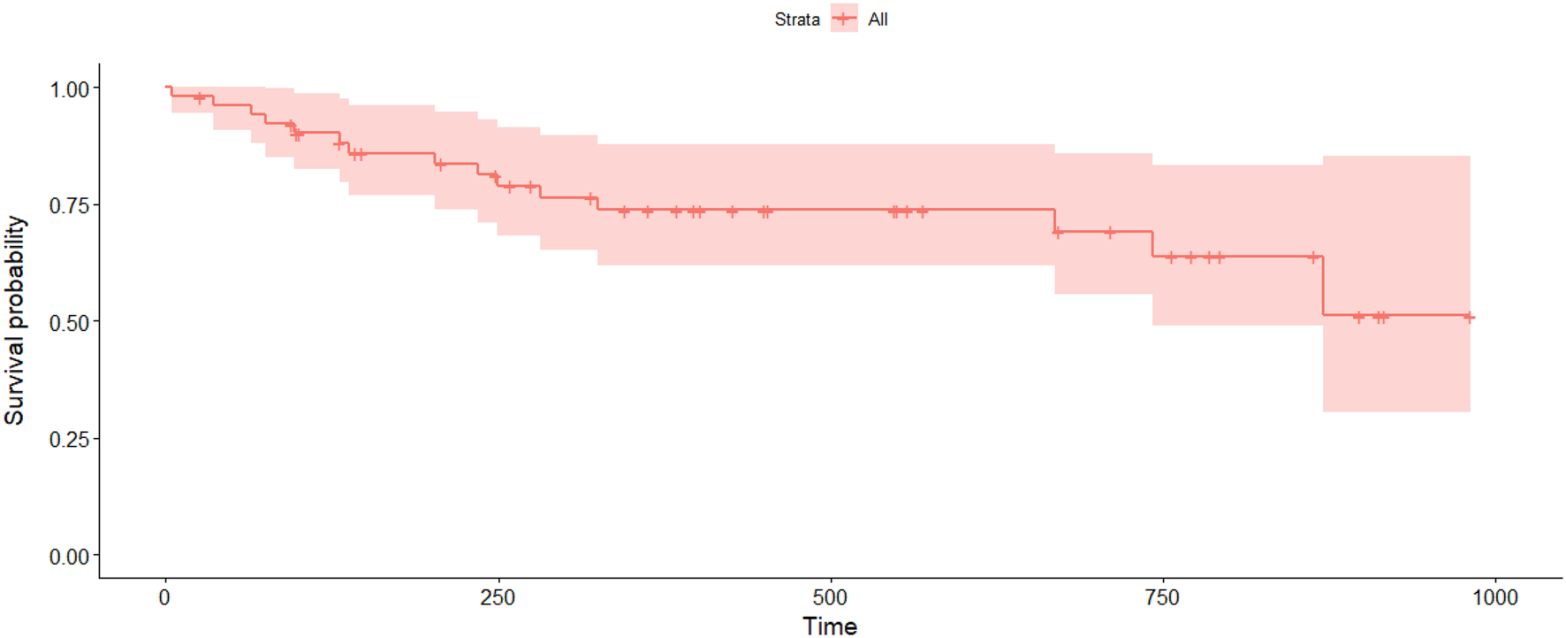
Curve of the overall survival probability of Eurasian lynx (N = 52) released in 2019–2021, estimated with the Kaplan–Meier method (time expressed in days; the area shaded pink represents the 95% confidence interval).

**Figure 4:**
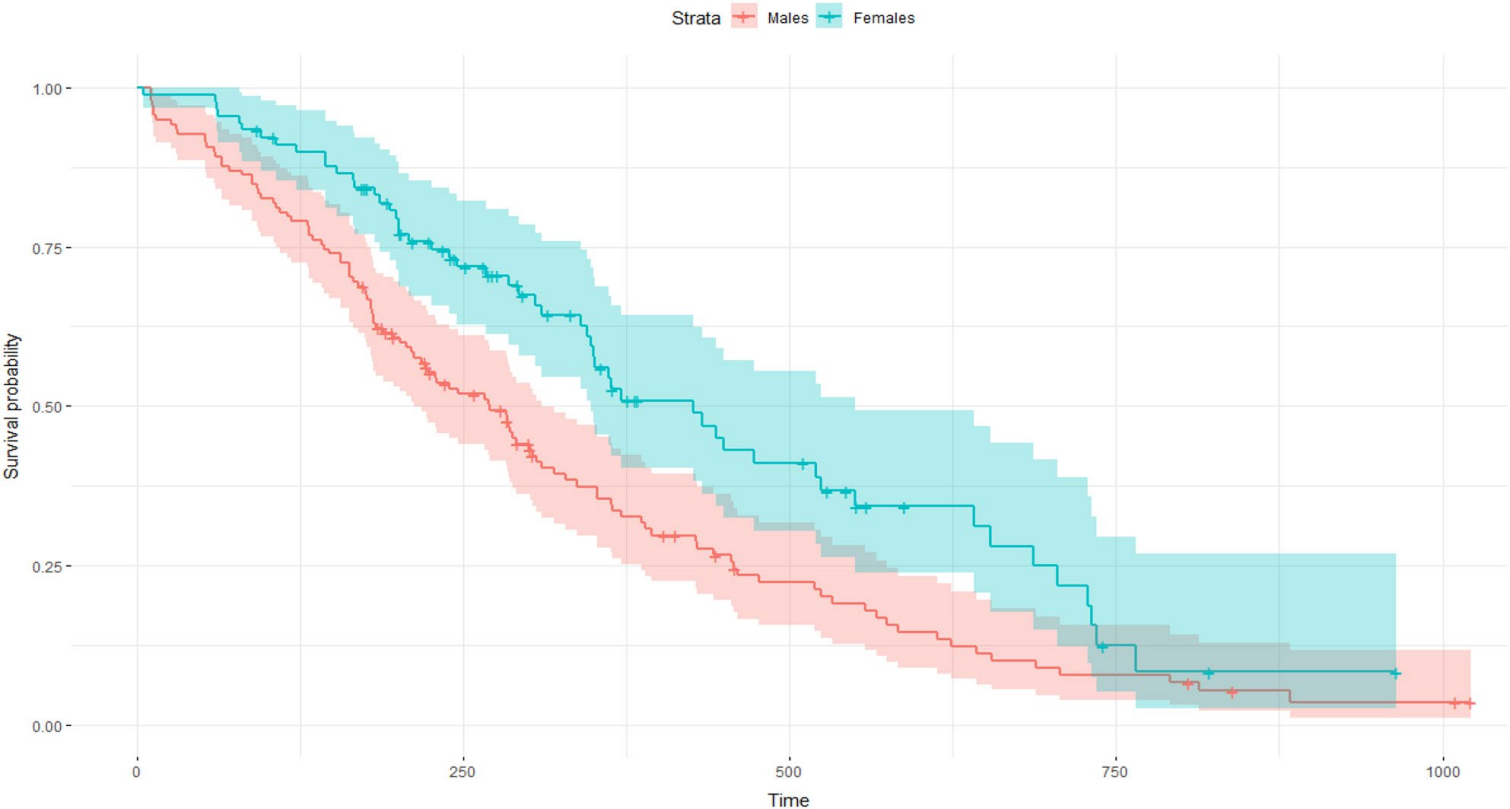
Survival curves for males (N = 28) and females (N = 24) of Eurasian lynx released in 2019–2021 (time expressed in days, shaded areas represent the 95% confidence interval).

Most mortality was recorded during the first 300 days following release (Figs. 3 and 4). The overall probability of surviving beyond three, 12, 18, 24 and 30 months was equal to 92.2%, 73.8%, 70.1%, 65.4% and 60%, respectively (N = 52). The survival time for males declined to a plateau of about 60% in 680 days, while the fall in female survival was slower, declining to a plateau of about 55% in 740 days.

**Figure 5:**
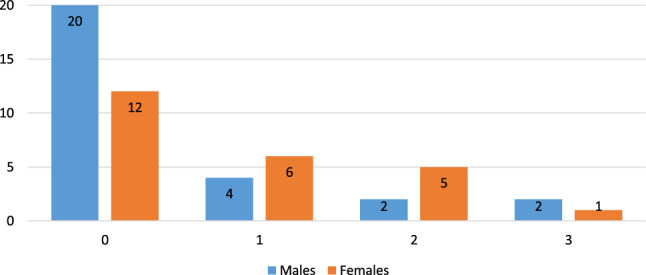
The number of mating periods that reintroduced, sexually mature Eurasian lynx survived in the wild from September 2019 to September 2021.

Taking into account the age of sexual maturity (3 years for males and 2 years for females^5^), the released animals survived in the wild for 1.65 mating periods on average, during which they had the opportunity to mate and breed (N = 20). For males (N = 8) this average was 1.75, and for females (N = 12) 1.58 mating periods (Fig. 5).

### Cox proportional hazard analysis

According to the Cox proportional hazard regression analysis (Table 2), the following variables were significantly related to survival—age at release (P = 0.0002, Hazard ratio = 0.718), training time (P = 0.0003, Hazard ratio = 1.011), defined as the number of days spent in adaptation enclosure and release centre, and year of release (P = 0.0117, Hazard ratio = 0.197) (N = 50). No evidence was found for any effect of sex, month of release or place of release.

**Table 2:** Results of the Cox proportional hazard regression analysis (N = 50; the value of estimate means the covariate increases the hazard of death/decreases the survival time; HR_95_ and HR^95^ indicate the lower and upper 95% confidence limits, respectively).

Covariate	Estimate	Standard error	Z	Hazard ratio	HR_95_	HR^95^	*P*-value
Age at release	− 0.332	0.090	− 3.678	0.718	0.602	0.857	0.0002
Sex	1.266	0.952	1.330	3.545	0.549	22.888	0.1836
Month of release	− 0.121	0.120	− 1.009	0.886	0.701	1.123	0.3130
Year of release	− 1.622	0.645	− 2.521	0.197	0.056	0.697	0.0117
Training time	0.011	0.003	3.589	1.011	1.005	1.017	0.0003
Place of release	− 0.225	0.360	− 0.626	0.798	0.395	1.616	0.5314

The significance of the proposed model was evidenced by the Likelihood ratio test (Statistic = 43.6, df = 6, P = 0.000), the Wald test (Statistic = 23.61, df = 6, P = 0.000), and the Score (logrank) test (Statistic = 29.31, df = 6, P = 0.000).

### Causes of death

**Figure 6:**
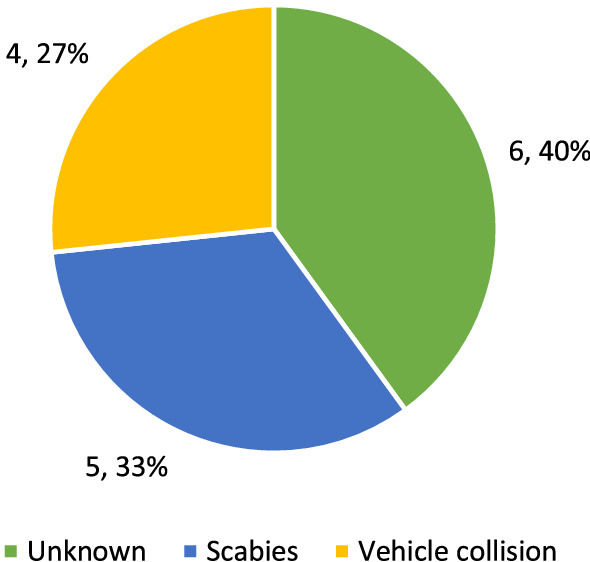
Mortality factors recorded for Eurasian lynx released as part of the reintroduction programme in north-western Poland (2019–2021).

The cause of death could not be determined in 6 out of 15 cases of mortality among the released animals recorded in 2019–2021 (Fig. 6). The majority of deaths (55.6%) were attributed to environmental factors, i.e. scabies (sarcoptic mange), and four (44.4%) were caused by humans (animals hit by a road vehicle or, in two cases, by a train).

There are indications that one of the animals may have been killed by a poacher, one may have died of an unknown infectious disease and one may have been killed by a predator (possibly another lynx). However, there is no hard evidence to substantiate these suspicions.

**Figure 7:**
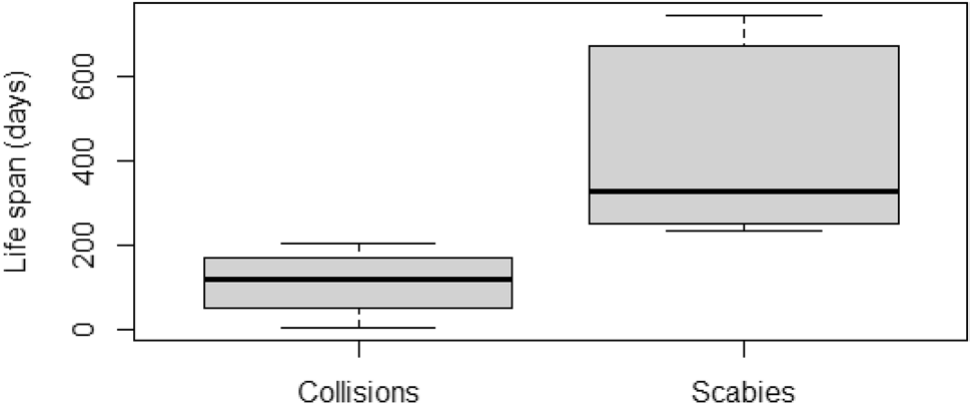
Cause of death of Eurasian lynx, released as part of the reintroduction programme in north-western Poland (N = 9), as a function of the time spent in the wild (the thick line inside each box indicates the median value, the edges of the box are the 25th and 75th percentiles, and the whiskers extend to the most extreme points).

There was a statistically significant difference in the life expectancy of lynx in the wild from the day of release to the day of confirmed death caused by a collision with a road vehicle or train and by other causes of death, most probably infection with environmental factors, like scabies (Mann–Whitney *U*-test, *P* = 0.015). This indicates that the limiting value to which death was caused by road and rail collisions is 200 days after release. After this period, environmental factors (scabies) was the main cause of death of animals in the wild (Fig. 7).

## Discussion

The success of a long-term reintroduction programme is assured when a viable, sustainable, ecologically and genetically functional population has been restored in the wild. The effectiveness of measures implemented to achieve this goal can be assessed with the aid of post-release monitoring, defined by indicators of short-term reintroduction success, i.e. survival of released animals and breeding by released animals^30^. The very effectiveness of these measures depends on the animals’ hunting efficiency (adaptation to life in the wild conditioned by pre-release training) and the avoidance of human-carnivore conflicts (appropriate reintroduction programme design and conflict management in the later stages), with subsequent mating that ends in parturition and the rearing of kittens (reproductive success)^31,32^.

On average, seven out of every ten lynx released in 2019–2021 in north-western Poland, whose fate is known, were alive as of 30 September 2021. This tallies with survival rates between 63 and 95% for this species reported by other authors (as revised by Franz and Romanowski^8^). The mean survival time (from release to recorded death) of animals released during the above-mentioned three-year period was 275 days (5–870 days). However, one male—still alive—has so far spent 981 days in the wild. This mean value is comparable with that for the reintroduced population in the Kampinos National Park in Poland (272 days)^8^. The average age of lynx released in north-western Poland is, as of 30 September 2021, slightly above 37 months. The Eurasian lynx is reported to live up to 17 years in the wild, but the average age of resident animals in a population is only about 4–5 years^5^. At the same time, it is estimated that under natural conditions, only 24% of lynx lived for three years in the Białowieża Forest (Poland)^33^. A study carried out in the Bohemian Forest, on the Czech-German border, indicated that individuals up to 4 years of age made up 64% of the entire population and that the mean generation time for resident reproducing females was 2.64 years^34^. Meanwhile, the probability of reaching the age of 2.5 years for lynx reintroduced to north-western Poland was 51.1%. The sex of the animals had no effect on the probability of survival, which suggests no effect of sexual dimorphism. Differences in the probabilities of male and female survival have been found among other carnivores, both in species with (e.g. European mink *Mustela lutreola*^35^) and without sexual dimorphism (e.g. swift fox *Vulpes velox*^36^).

When assessing the reproductive success of the reintroduced lynx, one has to assume that the number of mating seasons during which they could reproduce is comparable with the number of litters, i.e. mating seasons during which animals successfully reproduced. In the case of the lynxes covered by the “Return of the Lynx to north-western Poland” project, ten litters were recorded in 2019–2021, which translates into 30.3% of the chances of mating and successful breeding. On the other hand, the fact that eight females had a litter at least once means that 80% of the sexually mature reintroduced animals (83.3% of females) reproduced at least once in the wild in 2019–2021. For comparison, only 35% of the released females in the population reintroduced to the Kampinos National Park were found to be breeding^37^.

The effect of the covariates on the survival curve was shown to be strong and significant for the age at release and the time spent in the adaptation enclosure and release centre (training time), while year of release had less significance. The hazard ratios of covariates, explaining the multiplicative effects on the ratio of death, indicate in particular that being older when released augurs well for survival in the wild, whereas a longer training time is linked with poor survival prospects. The results therefore suggest that the post-release survival of younger reintroduced individuals is lower, probably because they have a lower potential for coping with the stress associated with translocation than older ones^38^. The relationship between the survival rate of reintroduced animals and the year in which they were released is governed by stochastic meteorological factors (obviously independent of humans) and therefore cannot be mitigated in the hard-release scenario. To overcome interannual variation as regards climate and infrequently occurring natural disturbances, IUCN recommends releasing individuals over several years^28^. It should also be noted that the number of releases in a given year may distort the interpretation of the results, as more releases in later years translates directly into fewer days spent in the wild by late-released animals. There is no evidence of any effect of sex, month of release and the place where the animals were released on the life expectancy of reintroduced lynx in the wild.

Both, sex of released individuals and the place of release (analysed in terms of linear distance dependence on the training centre) makes no statistically significant contribution to the difference in the hazard ratio after adjusting for age, sex, month, year and training time. In the case of lynx reintroduced to north-western Poland, releasing the animals in the earlier months of the year did not appear to favour their greater survival. Such a dependence was demonstrated by the results of an experiment demonstrating that the survival of released least weasels *Mustela nivalis* improved when releases took place during a season with abundant food resources^39^. The Canada lynx *Lynx canadensis* reintroduction protocol also recommends releasing them in the spring months, so to ensure the highest annual abundance of prey^40^. The insufficient amount of data probably determined the lack of significance of survival in relation to a place of release, determined by the distance from the training centre. Despite some tendencies reported by practitioners, this issue requires further investigation.

According to Maran et al.^35^ the following factors potentially influence the survival of released animals: biological variables (age, sex), pre-release factors (maintenance conditions, pre-conditioning, experience with humans), release methodology (hard or soft release), release site characteristics (availability of suitable shelter and habitat, abundance of predators, availability of food resources, level of disturbance). The biological variables include the genotype and thus the origin of the animals. The use of wild animals in mammalian carnivore restoration is sometimes claimed to be more successful than the use of captive-born ones^38,39^. In contrast to the above statement, the use of captive-breed Eurasian lynx is recommended for reintroductions^41^. The lynx recruited for the reintroduction programme in north-western Poland were of captive origin and belonged to the Baltic population. Two aspects favour this approach. Firstly, the reintroduction carried out in the Harz Mountains (central Germany) demonstrated that zoo-born individuals were able to adapt to life in the wild, and secondly that a considerable advantage of captive-bred animals over wild ones is the possibility of conditioning their genetic profile and health status before release^41^.

An analysis of previous restitutions of mammalian carnivores points out that far too little attention is paid to sociological and organizational variables^20,42^. The latter include pre-release treatment, behavioural conditioning (training) and pre-release housing conditions (active adaptive management)^28,35,43^. As it turns out, the previously mentioned claim regarding the greater survival chances of reintroduced wild-born animals is based largely on the fact that they are better able to tolerate translocation stress^38^. At the same time, many authors emphasize the possibility of a positive impact of appropriate pre-release training (learning effect) on shaping the behaviour of individuals intended for reintroduction (e.g. inducing development of “natural” behaviours, familiarity with the natural habitat and avoidance of danger), increasing their post-release chances of survival and life span^8,28,30,38,44,45^. The results indicate that pre-release training (enclosure effect) significantly influences the survival of reintroduced animals, but in a rather non-obvious way: remaining too long in the adaptation enclosure and the release centre is negatively correlated with the number of days that the released animal survives in the wild. While the training itself is undoubtedly of great importance in adapting to post-release life in the wild, too much of it can produce a side effect in the form of pre-release habituation to captivity, to humans and, generally, to stimuli that may pose a danger after release, as has been shown, for example, in the case of released swift foxes in North America^44^.

The mortality of reintroduced animals is a derivative of biological and sociological variables. It is commonly believed that most lynx mortalities in Europe are caused by humans and result from recreational harvesting, poaching and vehicle collisions^5,8,20,46,47^. In the reintroduced population in north-western Poland, the only confirmed causes of animal death were environmental factors (scabies) and traffic collisions. Moreover, the numbers of the former, i.e. death from a natural cause, were slightly higher. Likewise, in a strictly protected area in the Bohemian Forest, lynx were not found to be strongly affected by human-related mortality^34^. By contrast, a study in the Swiss Alps and the Jura Mountains (Switzerland) showed that natural causes accounted for 44.9% of all established causes of death in this region and that infectious diseases accounted for 26.5% of them. Scabies alone accounted for only 6.1% of deaths from a known cause^48^. Sarcoptic mange, caused by *Sarcoptes scabiei*, is a highly contagious epizootic skin parasitosis, distributed worldwide and observed most frequently in mammals^49^. Occasional cases of scabies as the cause of death of lynx in Europe have been reported from Norway^50^, Sweden^51^, Switzerland^52^ and Poland^49^. The source of infection in lynx is likely to be the red fox *Vulpes vulpes*, in which case *S. scabiei* must be endemic in some parts of Europe^52,53^. The recently upsurge in reports of outbreaks in new locations suggest that this parasite is expanding across the continent^53^.

One of the most interesting conclusions from the results of the present study is the clear distinction in life expectancy of reintroduced lynx that died as a result of traffic collisions and as a result of environmental factors, i.e. scabies infection. These results indicate that the former is the most serious cause of death in newly released animals (up to 200 days in the wild), while the latter represents the main known cause of mortality in animals surviving more than 200 days in the wild. This fact may be of great practical importance in terms of modifying pre-release training, but the topic needs to be further explored. Even if “quick learners” cannot avoid the natural, objective threat of scabies, their ability to avoid traffic accidents does enhance their chances of survival in the wild. Numbers of observations being limited, it is recommended that further data should be collected on lynx mortality and that analyses in this respect should be continued. It should also be borne in mind that appropriate pre-release training to avoid danger can have a considerable effect on lynx survival, as well as the implementation of conservation measures improving the safety of wild animals vis-à-vis road and rail infrastructure, such as under- and overpasses, reduced speed zones, and fencing^8^. The effectiveness of such an approach has been demonstrated in reducing the effect of road mortality on the Iberian lynx *Lynx pardinus* in Spain^54^, and the need for vehicle strike mitigation in the case of the bobcat *Lynx rufus* in Ohio (U.S.A.)^55^.

The results obtained for north-western Poland (in particular, the lack of significant human-lynx conflict cases revealed by the three-year post-release monitoring) augur well for the possible coexistence between lynx and humans, also reported from other parts of Europe, thus indicating that they can share the same landscape^7,56–58^. Cases of felids capable of persisting even in heavily human-modified landscapes (*Panthera tigris*, *Panthera pardus*, *Puma concolor*) were reviewed by Bouyer et al.^56^.

In conclusion, although the present study was to some extent limited by the sample size, the short-term results of the Eurasian lynx reintroduction programme in north-western Poland provide the most comprehensive and substantial scientific basis to date for planning the restoration of this felid in the Southern Baltic Lake District, and also inform future releases of this felid in similar conditions. The following conclusions and recommendations can be formulated, based on the results obtained and literature review:

1. In order to ensure better survival, it is preferable that mature animals be released.
2. The pre-release training of animals should not last too long and should focus on arousing fear in them of road vehicles and trains.
3. As the first 300 days after release are critical for the release operation (the period with the highest mortality), any interventions supporting the survival of the released individuals should focus on this period.
4. Telemetry collars make it possible to precisely monitor the fate of the released animals, thus enabling a comprehensive assessment of the short-term effectiveness of the reintroduction measures.
5. Monitoring of the reproduction, movements and behaviour of the released animals should be continued (telemetry collars replacement and maintenance) in order to assess the long-term effectiveness of the reintroduction project.

It should be also noted, that despite the promising results regarding the survival and reproduction rate of the released animals, it seems advisable to continue the reintroduction project of the Eurasian lynx in north-western Poland at least until the number of kittens born in the wild begins to compensate for the losses due to sub-adult and adult mortality. Termination of the project at the present stage would mean the gradual disappearance of the population hitherto created, as shown by the experience from central Germany^19^. However, further research into the reproductive success of the reintroduced population is necessary.

## Methods

### Study area

**Figure 8:**
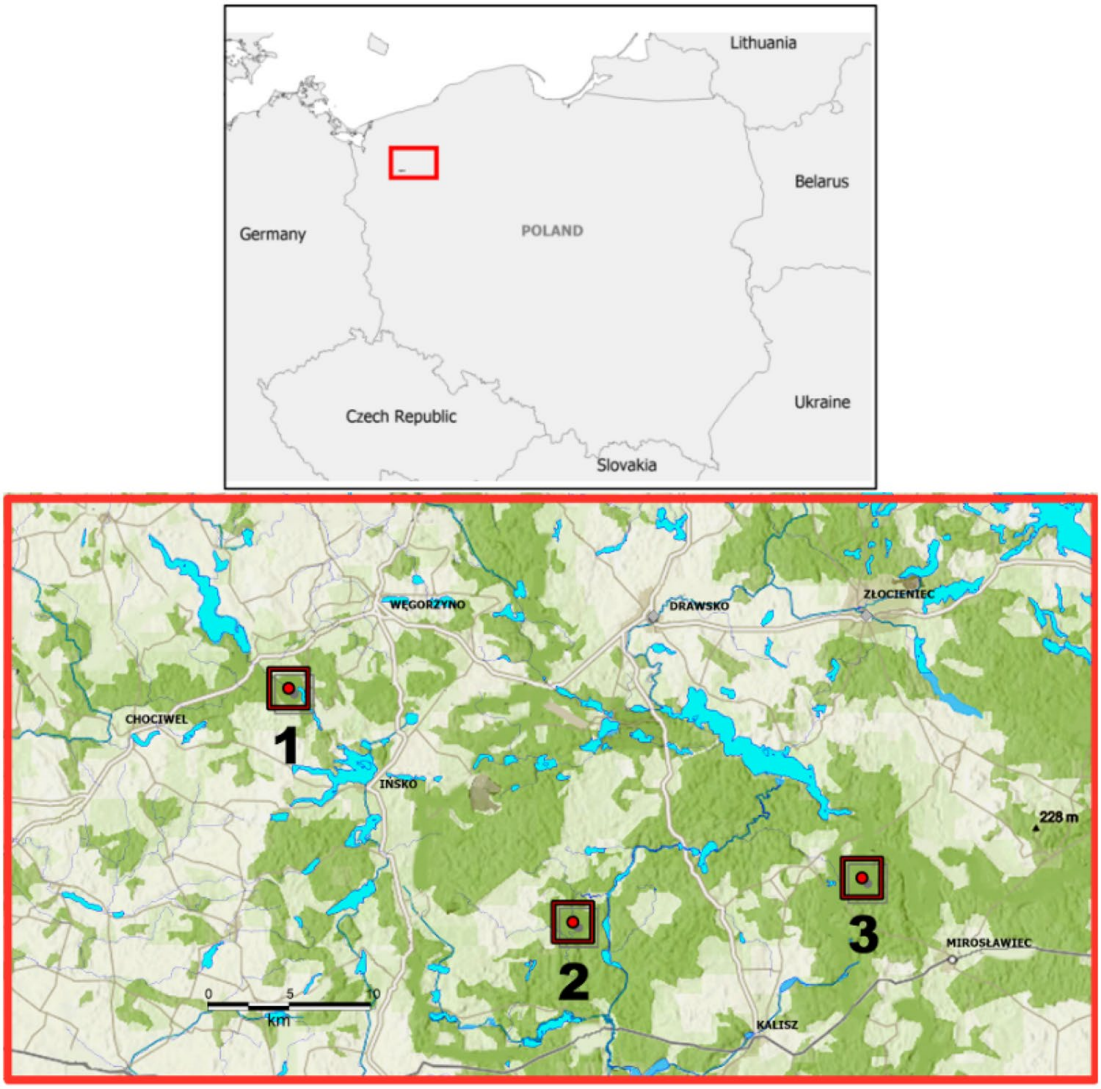
Release sites for the Eurasian lynx reintroduction programme in north-western Poland (1—adaptation enclosure in Dłusko, Ińsko Lakeland, 2—release centre in the Drawsko Forest District, 3—release centre in the Mirosławiec Forest District). Map developed in MapInfo Professional v11.0 (PitneyBowes, U.S.A.).

The area selected for reintroduction (Ińsko Lakeland, Drawsko Military Compound and Mirosławiec Forestry; Fig. 8) is situated in the Southern Baltic Lakeland (province of Western Pomerania). It is a young post-glacial landscape, well forested (44% of the area is woodland), with minimal fragmentation (a dense network of forested ecological corridors) and large numbers of roe and red deer (720 kg/km^2^)^26^. Tree stands more than 100 years old account for c. 10% of the forest area^59^. The dominant tree species is pine (nearly 60% of all the forest trees), and the amount of harvested timber is 572.1 m^3^ per 100 ha of forest^59^. The average annual air temperature is 10.8 °C, with extremes of − 30 and 37.8 °C. The total annual precipitation is 705 mm, and the averages of wind speed, insolation and cloudiness are 3.5 m/s, 1.976 h and 4.9 octants, respectively^59^. 21.8% of the area consists of land subject to various forms of legal protection (national parks, nature reserves, landscape parks, etc.)^59^. The region is predominantly rural (93.8%), with an average population density of 74 inhabitants per km^2^. At the same time, 68.4% of the population lives in urban areas^59^. The densities of main roads, secondary roads and railways are 0.08, 0.10 and 0.05 km/km^2^, respectively^26,59^. There are 0.78 road vehicles per inhabitant^59^. It is estimated that the project area can host at least 80 lynx, guaranteeing a survival of 100 years at the level of 57% if isolated^26,60^.

### Origin of the released animals

The Eurasian lynx reintroduction programme in north-western Poland involved 61 captive-born individuals originating from the Baltic population, imported from European zoos and enclosures (not associated with the European Association of Zoos and Aquaria, EAZA), and two animals born in the Dłusko breeding centre (Table 1)^26,27^. Individuals kept in this breeding centre derive from the EAZA breeding programme, for which a European studbook (ESB) for Northern lynx *L. l. lynx* was established. Only individuals that genetically did not differ significantly from wild lynx within the Baltic population were included in the breeding and reintroduction programme (each individual was subjected to genetic testing prior to release).

### Release methodology

The reintroduction was carried out within the framework of the EU-financed project “The Return of the Lynx to north-western Poland”. The conditions for reintroduction were laid down in the decisions of the General Directorate for Environmental Conservation (DZP-WG.6401.08.10.2017.JR.bp of 2017, DZP-WG.6401.08.2019.bp of 2019, and DZP-WG.6401.08.46.2019.TŁ of 2020) and followed IUCN guidelines^28^. All experiments were performed in accordance with the Polish nature protection law, regulating the proceedings (restrictions and prohibitions) with wild animals subject to legal protection in the territory of the Republic of Poland—Act of April 16, 2004 on nature protection (Journal of Laws of 2004, No. 92, item 880). After quarantine and acclimatization, during which the lynx were carefully monitored (photo-traps, CCTV system, direct observation during feeding), they were assessed for the next pre-release steps. The period of adaptation was individually adapted to the behaviour of each animal. Most lynx required only a short, 1–2 week training period before release, but some others needed more training and stayed longer in the semi-natural environment of the adaptation enclosure in Dłusko. The Dłusko facility has six enclosures from 0.5 to 0.8 ha in size, located within a fenced area (enclosure) of 90 ha. Careful observation made it possible to select lynx for breeding, which were then moved to the breeding centre in Jabłonowo (facility of the West Pomeranian Nature Society) because of their low degree of anthropophobia. Secretive animals, exhibiting shy, cautious behaviour and avoiding contact with humans, were intended for release and trained for hunting wild prey. It should be noted, however, that the lynx's strong hunting instinct does not require reinforcement and the main behaviours to be enhanced were avoidance of humans and arousing fear of humans^26,27^. Their contacts with keepers were thus reduced to a minimum and restricted to unavoidable husbandry. During the entire pre-release training period, the animals were fed exclusively with the meat of their natural prey. Lynx intended for release were moved to the outside enclosures of the release centres in Mirosławiec and Drawsko (situated in forests), where they were initially fed, after which feeding was limited and the lynx were left outside the enclosure, usually after 1 week. During adaptation period animals were kept in natural enclosures, with trees, bushes, fallen trees, branches, stones and several hiding places (in the case of the adaptation enclosures in Dłusko, and release centres in Mirosławiec and Drawsko; Fig. 8). Related individuals were released in different locations, except for mothers and their kittens. Releases took place in different months throughout the year, in forested areas away from human settlements, and followed a hard-release protocol. The age, sex, release date and place, and time spent in the adaptation enclosure and release centre were recorded for every individual released. Due consideration was given to animal welfare at all stages of the reintroduction process.

### Radio-tracking and post-release monitoring

All released lynx were tracked using GPS telemetry. Each released individual was fitted with a GPS/GPRS/VHF telemetry collar weighing from 280 to 320 g, matched to the animal’s size. The collars, manufactured according to an original design by the West Pomeranian Nature Society, have a GPS locator that records a lynx’s position every 3 h, a GPRS/GSM communication module that transmits data to telemetry servers twice a day, and a continuously operative VHF transmitter with an independent power supply, enabling the animal in question to be sought in the field. The maximum estimated working time of the GPS module is 15 months, whereas the VHF transmitter can work for up to 3 years. The collar is equipped with a mechanical dropper, which allows a lynx to lose its collar after about 2–2.5 years. Collars with dead batteries were replaced with new ones. All the antennas are concealed in the telemetry device case and collar strap, as a result of which the collar has no protruding components. With telemetry data one can determine with a high degree of probability the sites where hunting has been successful. In the first period after release, all the sites where prey was caught were checked to assess the degree of independence of the released animals and the type of catch. In the following months, all lynx that exhibited atypical movements or were inactive (stationary) for a suspiciously long time were tracked and monitored in the field. Where necessary, on site observations enabled the cause of unusual behaviour or death to be discovered. Individuals lost to radio-tracking and not subsequently recaptured were assumed to have died (designated as "unknown status" and excluded from further analysis).

Post-mortem examination was conducted by a qualified veterinarian, in accordance with routine veterinary medicine protocols and post-mortem procedures for wildlife veterinarians^61^, on all dead lynxes found in the field, in order to determine a proximate cause of death. The examination included a systematic necropsy, except in cases where obvious traumatic traffic accidents caused the death (X-ray imaging performed if needed), and was focused on the following etiological factors: congenital, infectious, nutritional, toxicological, traumatic, other potential reasons founded on generalized etiologic principles (politraumatic events, multi-raility of limbs, fractures, traumatic haemorrhage). Scabies was diagnosed based on a visual observation of skin lesions and confirmed by detecting the mites in skin scrapings with microscopy. Mites were collected by scraping affected skin areas with a sterile surgical blade, following by placing samples on a glass plate, dripped with liquid paraffin and consequently covered with a glass cover and then examined microscopically under 100–400 × magnification. Mites were morphologically identified as *S. scabiei* using a diagnostic reference^62^. The submission of tissue samples for further diagnostics was based on necropsy findings when additional insight was warranted to confirm or determine the cause of death, and included microbiological (bacteriological culture), biochemical (c-reactive protein) and toxicological (coumarin and coumarin derivatives, ethylene glycol, strychnine, organophosphorus insecticides, bromethalin, petroleum, toxin alkaloids, cicutotoxin, organochlorine insecticides and mycotoxins) analyses commissioned to reference laboratories. During the necropsy examination all samples were taken in rigorous asepsis conditions and transported for the further analyses as soon as possible. All post-mortem examinations were documented (descriptively and photographically).

### Survival analyses

The survival rate was calculated as the percentage of individuals remaining alive as at 30 September 2021 in the pool of animals released into the wild and not caught and placed in the breeding centre, and whose fate in the wild was known (N = 52). Lynx whose fate in the wild was not known were included in the analysis of hypothetical survival rates calculated on the assumption that these animals were alive or dead (N = 59). Life expectancy was calculated for those animals whose death was recorded after release into the wild (N = 15), to exclude the effect lowering the value of this indicator, resulting from the analysis of the situation for the selected time point (30 September 2021), after which a significant percentage of the released animals are still alive. In addition, the average number of mating periods survived in the wild by reintroduced sexually mature individuals was calculated.

Overall (including plot as a function of place and year of release) and sex-dependent cumulative survival probability curves were plotted by the Kaplan–Meier method^63^ and visualized using the ‘Survival’ v. 3.2-13^64^, ‘Survminer’ v.0.4.9^65^ and ‘SurvMisc’ v. 0.5.5^66^ packages for R^67^. Two males caught after being released and placed permanently in the breeding centre (Nos. 1 and 2 in Table 1) were excluded from the survival analyses (N = 59).

Statistica v.13.0 PL software (TIBCO Software Inc.) was used to assess the statistical significance of the difference in overall survival times between males and females (Mann–Whitney *U*-test for comparisons of median values)^62^ and differences in life expectancy of lynx in the wild from the day of release to the day of confirmed death caused by a traffic collision or by environmental factors, i.e. scabies (Mann–Whitney *U*-test)^68^. Statistical significance was at P ≤ 0.05.

### Measure of dependence among co-variables

Multivariate Cox proportional hazard regression^69,70^ was applied to investigate the association between the survival time (life expectancy) of released individuals and the following predictor variables: age (at release), sex, month of release, year of release, training time and place of release. For this, the ‘Survival’ v. 3.2-13 R package was used^64^. The significance of the proposed model was assessed by the Likelihood ratio test, the Wald test and the Score (logrank) test^71–73^. Two males placed permanently in the breeding centre (Nos. 1 and 2 in Table 1), two females caught after release and re-released in other locations (Nos. 11 and 28 in Table 1), as well as seven individuals of unknown status (Nos. 4, 25, 30, 37, 39, 41 and 53 in Table 1) were excluded from the analysis of the above-mentioned co-variables. All data used in the Cox analysis concerning live individuals at the time of closing the analysis were treated as a right-censored data. Sex data, of a binary nature, were coded as: males = 1, females = 2. In the case of data on a release site, the model uses the code 1 for animals released in an immediate vicinity of the training site, while the following numbers encode the two remaining release sites, the sequence of which is determined by an increasing distance to the training site: adaptation enclosure in Dłusko = 1, release centre in the Drawsko Forest District = 2, release centre in the Mirosławiec Forest District = 3 (Fig. 8).

### Ethical statement

The research presented in this study was conducted on the basis of a permit issued by the General Directorate for Environmental Conservation (DZP-WG.6401.08.10.2017.JR.bp of 2017, DZP-WG.6401.08.2019.bp of 2019, and DZP-WG.6401.08.46.2019.TŁ of 2020). All experiments were performed in accordance with the Polish nature protection law, regulating the proceedings (restrictions and prohibitions) with wild animals subject to legal protection in the territory of the Republic of Poland—Act of April 16, 2004 on nature protection (Journal of Laws of 2004, No. 92, item 880).

## Data Availability

All data generated or analysed during this study are included in this published article.
